# Land Use Explains the Distribution of Threatened New World Amphibians Better than Climate

**DOI:** 10.1371/journal.pone.0060742

**Published:** 2013-04-16

**Authors:** Fernanda Thiesen Brum, Larissa Oliveira Gonçalves, Laura Cappelatti, Marcos Bergmann Carlucci, Vanderlei Júlio Debastiani, Elisa Viana Salengue, Guilherme Dubal dos Santos Seger, Camila Both, Jorge Sebastião Bernardo-Silva, Rafael Dias Loyola, Leandro da Silva Duarte

**Affiliations:** 1 Programa de Pós-Graduação em Ecologia, Universidade Federal do Rio Grande do Sul, Porto Alegre, Brazil; 2 Programa de Pós-Graduação em Zoologia, Pontifícia Universidade Católica do Rio Grande do Sul, Porto Alegre, RS – Brazil; 3 Departamento de Ecologia, Universidade Federal de Goiás, Goiânia, GO - Brazil; University of Toronto, Canada

## Abstract

**Background:**

We evaluated the direct and indirect influence of climate, land use, phylogenetic structure, species richness and endemism on the distribution of New World threatened amphibians.

**Methodology/Principal Findings:**

We used the WWF’s New World ecoregions, the WWFs amphibian distributional data and the IUCN Red List Categories to obtain the number of threatened species per ecoregion. We analyzed three different scenarios urgent, moderate, and the most inclusive scenario. Using path analysis we evaluated the direct and indirect effects of climate, type of land use, phylogenetic structure, richness and endemism on the number of threatened amphibians in New World ecoregions. In all scenarios we found strong support for direct influences of endemism, the cover of villages and species richness on the number of threatened species in each ecoregion. The proportion of wild area had indirect effects in the moderate and the most inclusive scenario. Phylogenetic composition was important in determining the species richness and endemism in each ecoregion. Climate variables had complex and indirect effects on the number of threatened species.

**Conclusion/Significance:**

Land use has a more direct influence than climate in determining the distribution of New World threatened amphibians. Independently of the scenario analyzed, the main variables influencing the distribution of threatened amphibians were consistent, with endemism having the largest magnitude path coefficient. The importance of phylogenetic composition could indicate that some clades may be more threatened than others, and their presence increases the number of threatened species. Our results highlight the importance of man-made land transformation, which is a local variable, as a critical factor underlying the distribution of threatened amphibians at a biogeographic scale.

## Introduction

The worldwide decline of amphibian populations has become one of the main priorities on the conservation agenda. Amphibians are protagonists in the current biodiversity crisis, with one third of species threatened with extinction risk [1]. Amphibian decline and extinctions are both geographically and taxonomically structured [1], [2]. Threats are concentrated among montane forest and stream associated species in the Neotropics and Australia/New Zealand. Such declines are often propelled by habitat loss and fragmentation, climate change, pollution, and infectious diseases [3] – all threats resulting from the exponential growth of human population [4]. Despite the global influence of humans in amphibian extinction, it is still uncommon to include land use to explain amphibian distribution at the biogeographical scale. Much more common, however, is the use of climatic variables, which have been considered the main drivers of broad scale diversity patterns [5].

Recently, Ellis & Ramankutty [6] reclassified the global land cover into “anthropogenic biomes” or “anthromes”, based on global maps of land use, land cover and human population density. Incorporating anthropogenic biomes into conservation models may reveal patterns that could be markedly different from the traditional perspective of natural biomes, and could integrate human activities into a single view of ecological system. Moreover, anthromes are tractable biogeographical units and offer a more refined way to include land-use changes in geographically broad conservation planning.

The current rate of biodiversity loss has challenged ecologists to develop predictive models which summarize important ecological and evolutionary processes and, most importantly, to provide recommendations for on the ground conservation action that can be readily assimilated by decision and policy makers [7], [8]. Current knowledge focuses on phylogenetic and functional diversity [9], [10]. Functional diversity represents the extent of functional differences inside a community [11], [12], while phylogenetic diversity adds the species evolutionary relatedness into the diversity measure [9]. Where conservation is concerned, phylogenetic and functional diversity are important biodiversity components, as they ensure ecosystem services [13] and represent the evolutionary history of the target group [9]. Considering that all the metrics of phylogenetic/functional diversity aim to synthesize the phylogenetic/functional information, other dimensions of biodiversity end up being neglected. Two areas could have the same phylogenetic/functional diversity, for example, but have a completely different species composition. However, extinction risk is not independent of species identity, evolutionary history and ecological requirements [14], [15]; thus, species sharing the same ecological traits and/or phylogenetic affinities may be more prone to go extinct. This suggests that phylogenetic composition, in particular, may be a crucial driver of threatened species distribution at broad spatial scales.

We evaluated the direct and indirect influences of climate, land use, phylogenetic structure, richness and endemism on the distribution of threatened amphibians across the New World using path analysis [16]. We analyzed three distinct scenarios of conservation urgency in order to verify if the drivers of threatened amphibian distribution are the same for different levels of threat.

## Methods

### Species Data and Amphibian Threat Categories

We analyzed the direct and indirect influence of climate, land use (*i.e.* the anthropogenic biomes), phylogenetic structure, species richness and endemism on the distribution of threatened amphibians throughout the New World ecoregions. From the 289 New World ecoregions described by Olson et al. [17], we selected 262 based on the availability of climatic and phylogenetic data. The ecoregions used here ranged from 628 to 1,900,000 square-meters area. The range database we used [18] contains the current amphibian species list occurring in each ecoregion. We compiled the presence or absence of 2472 amphibian species in each ecoregion in a composition matrix W. We then obtained the species richness and the number of endemic species for each ecoregion. Species were classified as endemic if they occur exclusively in one ecoregion. Species richness and endemism were used as independent predictors of threat distribution in the path analysis (see below).

We classified amphibian species following the extinction risk categories proposed by the IUCN Red List Categories and Criteria [19]: Least Concern (LC), Near Threatened (NT), Vulnerable (VU), Endangered (EN), Critically Endangered (CR), Extinct in the wild (EW) and Extinct (EX). For each ecoregion, we calculated the number of species in each category. We ran our analysis based on three different scenarios: (1) the urgent scenario, containing only CR species and those EW and EX, (2) the moderate scenario containing all EN species and those at threat categories higher than EN (i.e. CR, EW, and EX), and (3) the most inclusive scenario, which included all VU species and those at higher threat categories.

### Land Use Data

**Figure 1:**
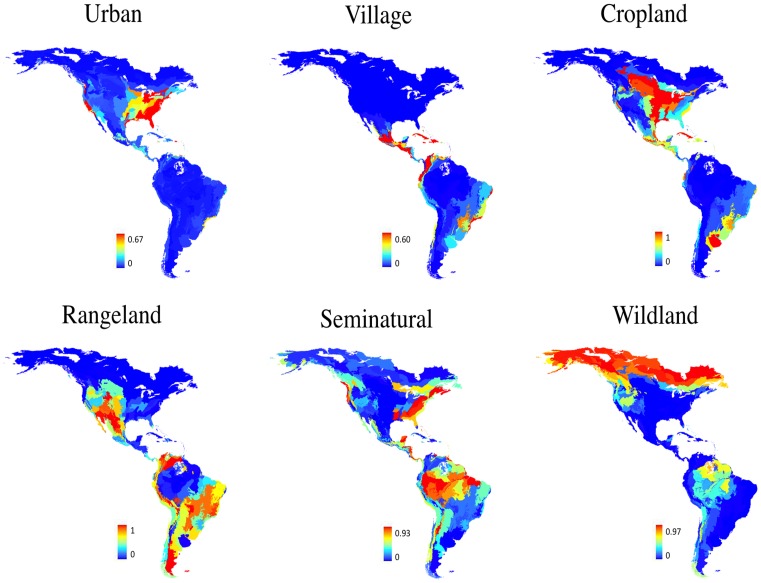
Maps depicting the spatial pattern of the proportion of land use cover in the New World ecoregions.

We used Ellis & Ramankutty’s [6] reclassification of global land cover based on land use and human population density. They named the new classification of the global land cover as anthropogenic biomes or anthromes. Using the zonal tabulate area tool in ArcGIS 9.3, we obtained the cover of each anthrome per ecoregion. In order to facilitate the interpretation of our results, we synthesized the cover of the 18 anthromes into six major categories, in decreasing order of human population density: urban (1788 persons/km^2^), villages (327 persons/km^2^), croplands (33 persons/km^2^), rangelands (7 persons/km^2^), seminatural (1person/km^2^) and wild (0 person/km^2^). The cover proportion of each anthrome category per ecoregion was treated as an independent variable in the path analysis (see below). The spatial distribution of the cover of different land uses along the ecoregions can be visualized in Figure 1.

### Climatic Data

To describe the climate in each ecoregion, we used nine environmental variables: altitude, annual mean temperature, temperature seasonality (standard deviation of temperature along the year × 100), maximum temperature of the warmest month, minimum temperature of the coldest month, annual mean rainfall, rainfall seasonality (rainfall’s coefficient of variation), precipitation of the wettest month and precipitation of the driest month. We decomposed each climatic variable into mean value and the range, totaling 18 climatic variables. All variables were compiled from the WorldClim 1.4 database [20], at the resolution of 2.5 arc-minute (∼5 km). Instead of using all nine variables in the analysis, we performed a principal components analysis in order to reduce climate complexity using the two first axes (climate axis 1 and climate axis 2), which concentrated 65% of all climatic variation, as descriptors. Correlations between climatic variables and climate axes are shown in Table 1.

**Table 1:** Correlation values of each climatic variable with the two first axes of the principal components analysis. M indicates mean values and R indicates range values.

	Climate 1	Climate 2
Altitude (M)	0.54	−0.56
Altitude (R)	0.46	−0.83
Annual Mean Rainfall (M)	−0.82	−0.28
Annual Mean Rainfall (R)	−0.49	−0.71
Annual Mean Temperature (M)	−0.87	−0.09
Annual Mean Temperature (R)	0.50	−0.82
Maximum Temperature of Warmest Month (M)	−0.63	0.25
Maximum Temperature of Warmest Month (R)	0.48	−0.80
Minimum Temperature of Coldest Month (M)	−0.88	−0.22
Minimum Temperature of Coldest Month (R)	0.56	−0.72
Precipitation of Driest Month (M)	−0.51	−0.07
Precipitation of Driest Month (R)	−0.49	−0.54
Precipitation of Wettest Month (M)	−0.82	−0.33
Precipitation of Wettest Month (R)	−0.47	−0.74
Rainfall Seasonality (M)	−0.03	−0.26
Rainfall Seasonality (R)	0.14	−0.63
Temperature seasonality (M)	0.75	0.45
Temperature seasonality (R)	0.75	0.02

### Phylogenetic Structure

To generate a phylogenetic tree of amphibians inhabiting the New World ecoregions we adopted the phylogenetic tree built by Pyron & Wiens [21].We fixed all branch lengths to unity. A phylogenetic pairwise distance matrix (D_F_) based on node counting for the genera contained in matrix W was computed using the software Mesquite 2.73 [22].

We scaled-up the phylogenetic relationships between species to the site level, generating a matrix describing the phylogeny-weighted genera composition of each ecoregion, which was defined using the phylogenetic fuzzy-weighting method developed by Pillar & Duarte [23], and implemented in the package SYNCSA-R [24]. For this, phylogenetic pairwise distances in D_F_ were used in terms of their complement as similarities (S_F_). Then, phylogenetic similarities in S_F_ were used to weigh the number of species per genera in matrix W. This procedure generated a matrix P containing phylogeny-weighted genera composition for each ecoregion. Accordingly, those j taxa most phylogenetically related to i (*e.g.* from the same genus) received a proportionally higher fraction of the presence of i in that ecoregion than more phylogenetically distant taxa (*e.g.* from a different genus), which will receive a proportionally lower fraction, and so on. Note that the sum of the number of species per genera (*i.e.* species richness) in an ecoregion belonging to W will remain exactly the same in P after phylogenetic fuzzy-weighting. Matrix P expresses the phylogenetic composition in the set of ecoregions.

By performing a PCoA [25] on matrix P, based on square-rooted Bray-Curtis dissimilarities between ecoregions [26], we generated principal coordinates of phylogenetic structure (PCPS, Figure S1). Each PCPS is a vector describing an independent phylogenetic gradient in the dataset [27]. The PCPS with the highest eigenvalue describes broader phylogenetic gradients related to the deepest tree nodes across the ecoregions, such as that connecting anurans and salamanders. As the eigenvalues of the other PCPS decrease, finer phylogenetic gradients related to higher nodes (e.g. families, genera) are described. PCPS analysis was done using the SYNCSA-R [24] and the package ape [28]. Then, the associations between amphibian phylogenetic clades and each phylogenetic vector were plotted in a correlation scatter plot.

### Path Analysis

To remove the effect of area and geographical position of each ecoregion, we did a set of multiple linear regressions between all the variables included in path analysis with latitude, longitude and area. Then, the residuals obtained from these regressions were used to build a causal model linking the different types of variables. Considering that, the final results of the analysis will represent the effect of climate, land use, phylogenetic structure, species richness, endemism on threatened amphibian distribution, with no influence of the area and geographical position of the ecoregions.

We evaluated the influence of the two climatic axes, six anthromes and the three phylogenetic filters in the distribution of threatened amphibians using model selection based on Akaike’s information criterion (AIC, [29]), separately for each group of variables, in order to select variables to be used as explanatory variables in path analysis.

Further, we evaluated causal connections between the selected climatic axes, anthromes, phylogenetic structure, species richness, endemism and threatened amphibian distribution using path analysis [25], [30]. The goal of this analysis is to evaluate the strength of causal relationships between more than two variables by decomposing the covariation between pairs of variables. We used the analytical approach proposed by Brum et al. 2012 [31].

**Figure 2:**
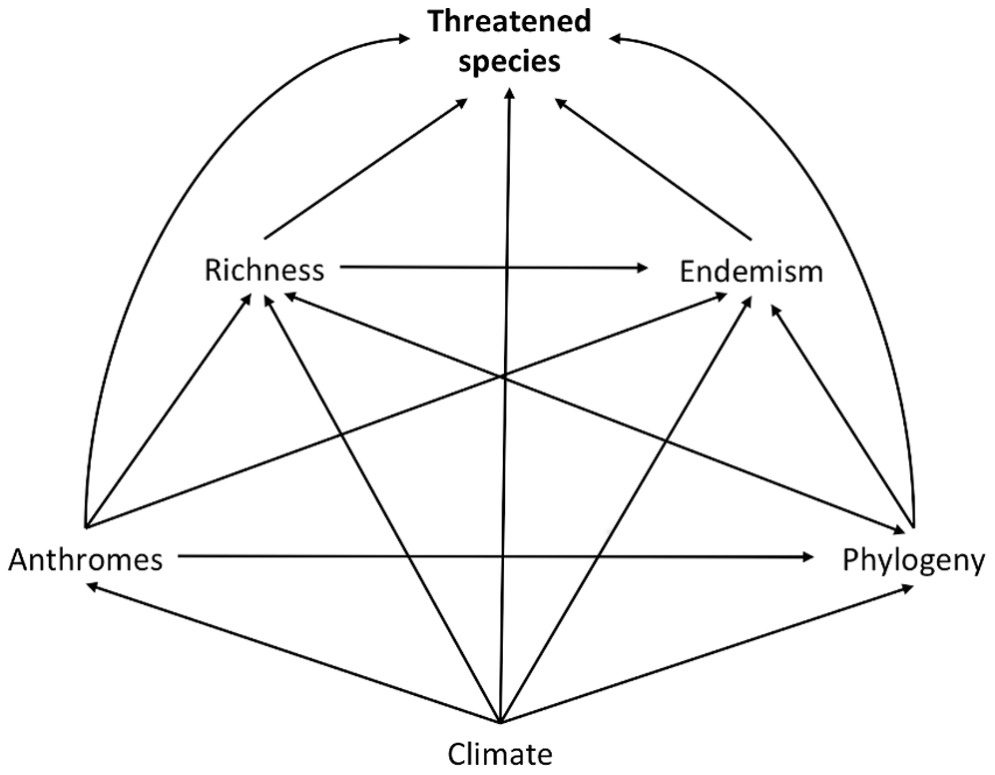
Hypothetical causal model establishing all possible and plausible causal connections between variables.

We built the path model in several steps using the model selection based on AIC. First, using the pre-selected climatic and phylogenetic variables plus species richness, endemism and threatened amphibian distribution, we built a hypothetical model establishing all possible and plausible causal relationships between variables (Figure 2). For this, a hierarchical causal order among explanatory variables was assumed. Climatic variables had the highest causal order, i.e. they are not determined by any other variable present in the model, also called exogenous [16]. All other variables were considered endogenous [16], since they could be determined by some other variable in the model (Figure 2). Threatened amphibian distribution had the lowest causal order, as it could not determine any other variable in the model (Figure 2).

Our second analytical step consisted in running a model selection to find which variables directly determined the variation in threatened amphibian distribution, based on AIC modeling. After that, we proceeded to iteratively find the explanatory variables determining each endogenous predictor of threatened amphibian distribution. That is, each variable found to determine threatened amphibian distribution was taken as a response variable, and their respective predictors were determined using model selection [31]. Thus, the final path model represented the best model connecting the variables causally structured according to our hypothetical model. We performed all the analytical steps separately for each scenario (urgent, moderate and most inclusive). Then, we obtained three final path models, one for each scenario.

We obtained path coefficients for the so-built models by linear multiple/simple regressions, being the standardized regression coefficient (β) equivalent to the path coefficient [16]. Since none of the variables were normally distributed (all failed in the Shapiro-Wilk normality test), the *P* values of each path coefficient were calculated by using randomization test [32]. Model selection procedures based on AIC were performed using the software SAM v4.0 [33] and simple and multiple linear regressions were performed using the software Multiv 2.4 [34].

## Results

**Figure 3:**
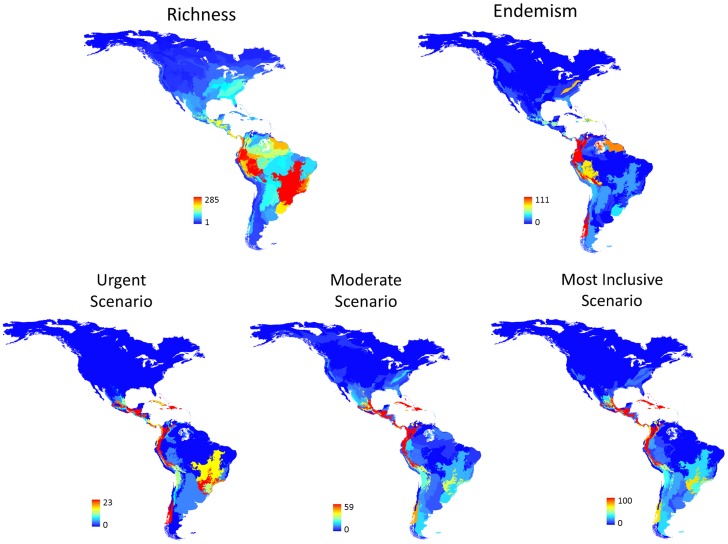
Maps showing the spatial pattern of richness of amphibian species, endemism and the number of threatened amphibian species according to the three different scenarios per ecoregion: the urgent scenario, containing only CR species and those EW and EX, the moderate scenario containing all EN species and those at threat categories higher than EN (i.e. CR, EW, and EX), and the most inclusive scenario, which included all VU species and those at higher threat categories.

**Figure 4:**
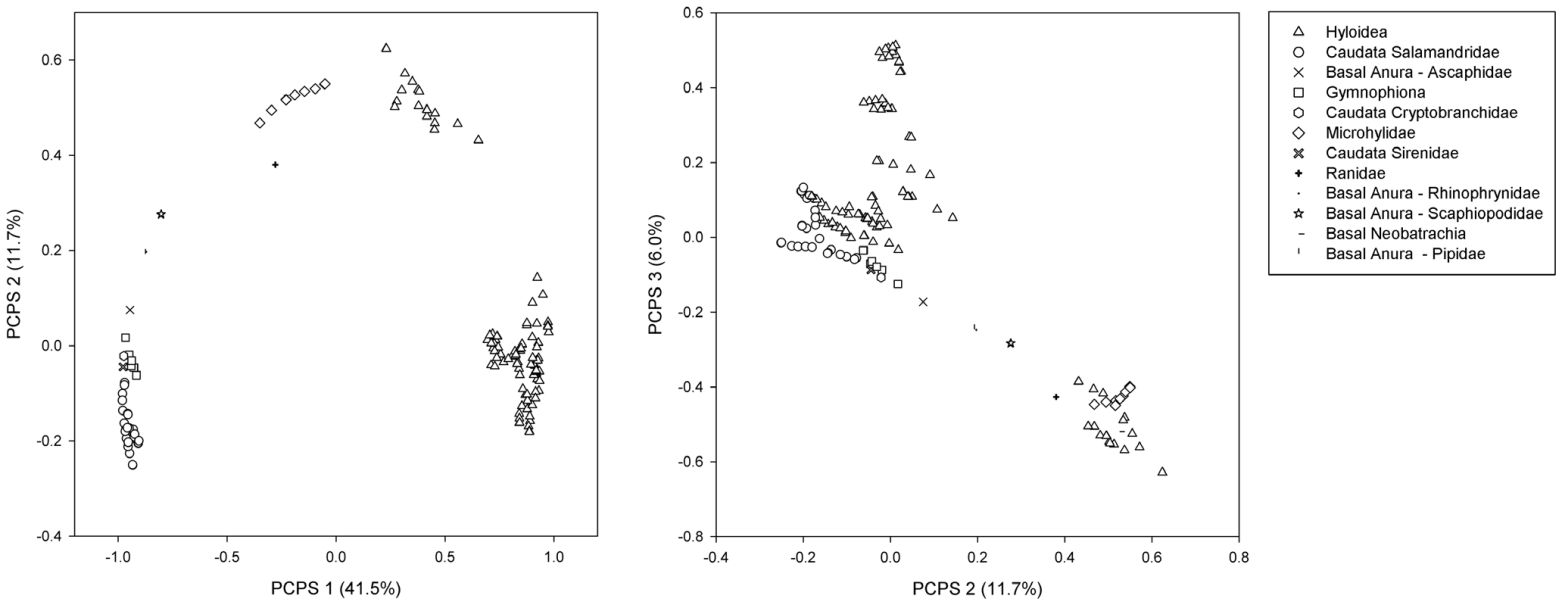
Correlation scatter plot for amphibian phylogenetic clades showing correlation values with three Principal Coordinates of Phylogenetic Structure (PCPS 1, PCPS2 and PCPS3) axes. Each point represents an amphibian genus. Genera are grouped within higher clades represented by different symbols.

From the 2472 amphibian species present in the ecoregions, 1886 belong to some threat category, 4 species were classified as Extinct, 221 as Critically Endangered, 326 as Endangered, 246 as Vulnerable. It means that the urgent scenario contained 225 species, the moderate scenario 551 species and the most inclusive 797 species. The maps showing the spatial distribution of species richness, endemism and the number of threatened species in each scenario is presented in the Figure 3 and the raw data could be visualized in the Table S1. Principal coordinate analysis for phylogeny-weighted species composition on matrix **P** generated 239 PCPS. The first three PCPS contained, respectively, 41%, 12% and 6% of the total variation of matrix **P**. Only the first three PCPS were submitted to model selection procedure, since most variation in phylogeny-weighted species composition (≅ 60%) was concentrated in these three orthogonal axes. The correlation of phylogenetic clades distribution and PCPS1, PCPS2 and PCPS3 is shown in the Figure 4.

**Figure 5:**
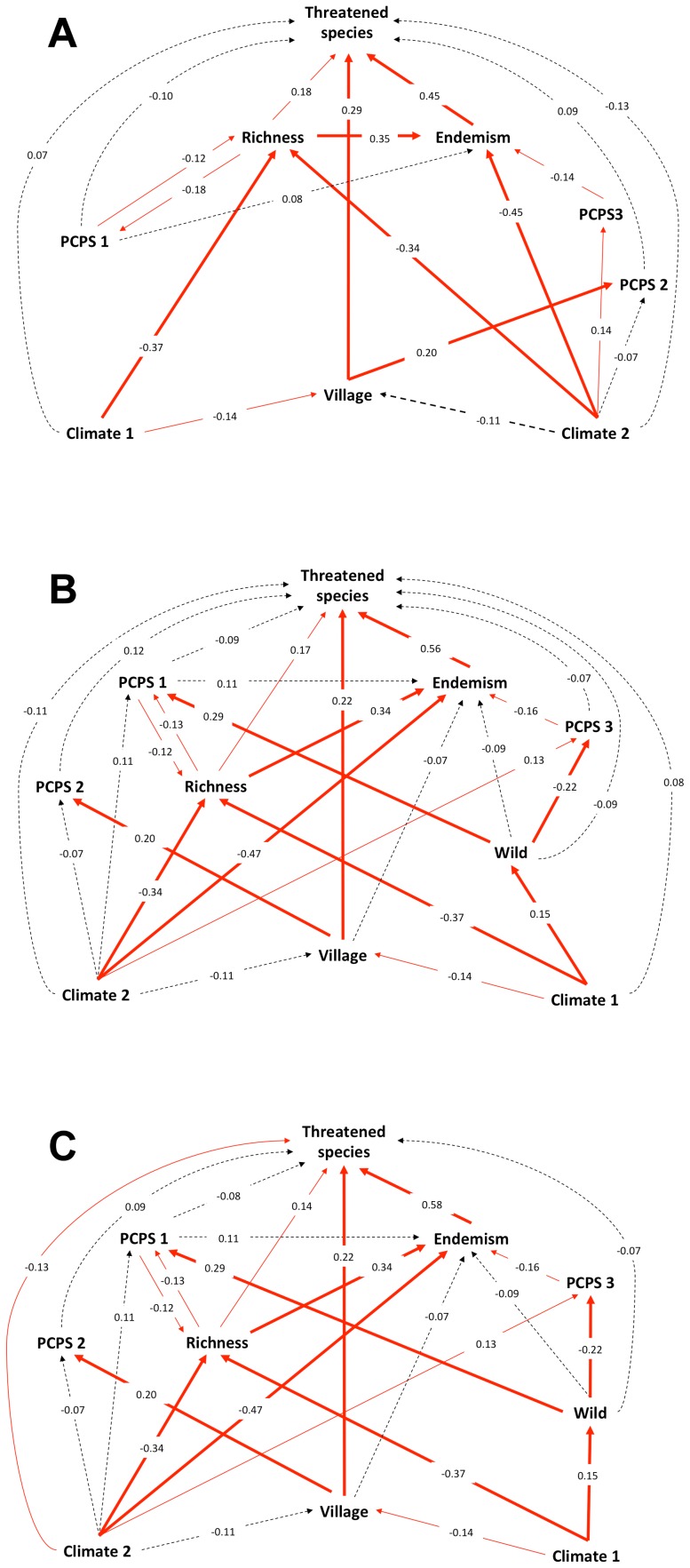
The final path model for the most urgent (a), the moderate (b) and the most inclusive (c) scenario, showing the causal relationships between climate axes (Climate) 1 and 2, proportion of village cover (Village) in each ecoregion, principal coordinates of phylogenetic structure (PCPS) 1, 2 and 3, amphibian richness (Richness) per ecoregion, the number of endemic species (Endemism) in each ecoregion and the number of threatened amphibian species in each ecoregion. Black dashed lines represent non-significant relationships between variables. Red lines represent significant path coefficients between variables and the line width represents the P value; narrow lines indicate to 0.05≥P≥0.01 and thick lines indicate P≤0.01. The path coefficients in the arrows are the standardized regression coefficients. The P values were obtained by randomization test.

Across the New World ecoregions, endemism was the best predictor in our urgent scenario, followed by the proportion of the village anthrome and species richness (Figure 5a). Phylogeny and climate were not important in directly explaining the number of CR and EX species in New World amphibians (Figure 5a), although they exert an indirect effect via species richness, endemism and land use.

In our moderate scenario, endemism, the village anthrome and species richness were also the main predictors of threatened amphibian distribution (Figure 5b). The moderate scenario model included a new anthropogenic variable (proportion of wildlands), which did not showed a direct influence on threatened amphibian distribution, but had an association with phylogenetic structure by strongly influencing PCPS1 and PCPS3.

The most inclusive scenario showed climatic factors as determinant of the threatened amphibian distribution, apart from the importance of endemism, proportion of villages and species richness variables (Figure 5c). The effect of endemism in the threatened amphibian distribution was greater than in previous scenarios. A correlation table presenting the correlation coefficients between all the predictor variables and the number of threatened species in each scenario is presented in the Table S2.

## Discussion

Despite the reported influence of climate on amphibian distribution [5], our models showed that the diversity components and anthromes are more important as direct predictors than the former one. Our results indicated that maybe climate and land use are acting in different time scales, with the climate operating in evolutionary time scales, influencing the richness, endemism and clade distribution of amphibians in the ecoregions. Now in the Anthropocene, when the current global extent of human transformation of ecosystems has already irreversibly altered the terrestrial biosphere [35], the conversion of wildlands to villages had a direct influence on the distribution of threatened amphibians. These results are straightforward and bring a sound message for amphibian conservation: the need to focus on land-use policies. Although scientists have long recognized and debated the direct and indirect effects of climate change on amphibian distribution, basing conservation actions upon such relationships may become a “Sisyphean task”. The feedback between climate and land use is well documented [36], [37]. Therefore, regulating land use may have direct effects on both amphibian extinction and climate change, and may be more feasible task than stopping climatic change.

Our results point toward a better outcome of amphibian conservation efforts if they are to be founded on land-use policies not only at the landscape level, but also at broader spatial scales. However, most current amphibian conservation actions are generally either species or site-specific. Our analysis has a particular caveat when applied to local actions. Although our results indicate that land-use change could drive diversity patterns not only at the landscape level [38] but also at the continental one, our analyses are too coarse to provide on-the-ground conservation support for local decision making. We believe, however, that our approach could act as a first filter to define guidelines for broad-scale conservation planning. Hence, when important regions are identified, our findings could be scaled down to sites within these regions, which would imply result in more manageable planning units [39].

It is largely known that human activities impact amphibian diversity [4]. Nevertheless, different types of land use likely determine distinct negative impacts on amphibian populations and, consequently, their extinction. The village land use category, which is more common in the developing world, synthesizes a variety of human activities, including agriculture and cattle grazing, in a densely populated context (village is the second most-populated anthrome category used in the present study) [6]. One in four people live in agricultural villages [6]. Pekin & Pjanowski [40] also found a negative influence of village settlements for some mammals groups, such as primates, bats and carnivores. An important aspect of our study is that we noticed a strong influence of land use, which is a landscape variable, on a broad scale biodiversity assessment. Thus, to assess the general causes of high levels of amphibian threat and extinction thoroughly, evaluations based on large samples and broad geographic scales are imperative [41].

Amphibian species with small geographical ranges are more prone to extinction than those with broad distributions, since they are more likely to be exposed to threatening process throughout their entire range, generally present a low abundance and they are often habitat or environment specialists [42]. Not surprisingly, the number of endemic species was the main factor increasing the number of threatened species, since they were defined as species occurring in only one ecoregion. The distribution of threatened amphibians was also indirectly associated with the presence/absence of some clades in the ecoregions, as both phylogenetic gradients, PCPS1 and PCPS3, showed significant associations with the species richness and endemism respectively. We found that the richness was higher in ecoregions characterized by all but Hyloidea clades, and the number of endemic species was higher in ecoregions characterized by the presence of Basal Anura, Basal Neobatrachia, Microhylidae, Ranidae and some families from Hyloidea clade. Considering that richness and endemism presented a positive relation with the number of threatened species, these finding suggests some degree of phylogenetic signal at the metacommunity level [23] in relation to the PCPS 1 and PCPS 3. Corey & Waite [2] found a strong signal of extinction threat within the amphibian phylogeny; the Hyloidea, a superfamily of frogs, includes more Critically Endangered species than any other clade in the amphibian phylogeny [2]. We found that the presence of some families of Hyloidea increases the number of endemic species and consequently the number of threatened amphibian species, corroborating with the patterns found by Corey & Waite [2].

Furthermore, land use was important not only via direct effects, but also through indirect effects by determining the spatial distribution of amphibian phylogenetic lineages. That is to say, closely-related species tended to be assembled by similar land use types, suggesting phylogenetic habitat filtering [27] in the geographic distribution of amphibian lineages. The positive relationship between cover of village and PCPS2 indicates that the Microhylidae, Basal Anura, Ranidae clades and some families from Hyloidea clade was more representative in areas with high population density and consequently intensive land use. The advancement of agricultural and colonization frontiers could be leading these clades to extinction, since the land conversion to this activities leads to the use of pesticides and other chemicals due to agricultural activities, and habitat loss as a consequence of forest conversion to pasture or croplands, all of these impacts known to cause amphibian decline and extinction [4].

In conclusion, our results showed that land use was directly more important than climate in determining the distribution of threatened amphibian species across the New World. Nonetheless, a considerable portion of the effect of land use on species threat was phylogenetically structured, meaning that human impact on amphibian distribution affects not only species individually, but may also define the fate of entire lineages of this imperiled group.

## Supporting Information

Figure S1 **Scaling-up of phylogenetic data from species to the site level employed in this study.** Matrices are: SF with phylogenetic pairwise similarities of species, Q′ is a transposed matrix with degrees of species belonging to every other species based on SF, standardized within columns, W with presence of species in sites, P with phylogeny-weighted species composition. Principal coordinates analysis of P using an appropriate dissimilarity measure generates a matrix of principal coordinates of phylogenetic structure (PCPS) composed of sites described by eigenvectors (EV). (Adapted from Duarte et al., 2012)
(TIF)

Table S1 **Raw data of richness of amphibian species, endemism and number of threatened amphibian species according to the three different scenarios (urgent, moderate and most inclusive) for each ecoregion, which were used in the analysis.** The urgent scenario, containing only CR species and those EW and EX, the moderate scenario containing all EN species and those at threat categories higher than EN (i.e. CR, EW, and EX), and the most inclusive scenario, which included all VU species and those at higher threat categories. The percentage values were calculated in relation to the total ecoregion richness.
(PDF)

Table S2 **Pearson correlation coefficient between the number of threatened amphibian species according to the three different scenarios (urgent, moderate and most inclusive), richness of amphibian species, endemism, two climatic axes, proportion of cover of villages and wildlands and the three axes of phylogenetic structure, all in residual form, which were used in the path analyses.**
(PDF)
