# Supplementary material for: Land Use Explains the Distribution of Threatened New World Amphibians Better than Climate
Source: PLoS One. 2013 Apr 16;8(4):e60742. doi: 10.1371/journal.pone.0060742 (PMC3628793; doi:10.1371/journal.pone.0060742)
Supplement: Table S1 — Raw data of richness of amphibian species, endemism and number of threatened amphibian species according to the three different scenarios (urgent, moderate and most inclusive) for each ecoregion, which were used in the analysis. The urgent scenario, containing only CR species and those EW and EX, the moderate scenario containing all EN species and those at threat categories higher than EN (i.e. CR, EW, and EX), and the most inclusive scenario, which included all VU species and those at higher threat categories. The percentage values were calculated in relation to the total ecoregion richness. (PDF) [file pone.0060742.s002.pdf]

Table S1. Raw data of richness of amphibian species, endemism and number of threatened amphibian species according to the three different scenarios (urgent, moderate and most inclusive) for each ecoregion, which were used in the analysis. The urgent scenario, containing only CR species and those EW and EX, the moderate scenario containing all EN species and those at threat categories higher than EN (i.e. CR, EW, and EX), and the most inclusive scenario, which included all VU species and those at higher threat categories. The percentage values were calculated in relation to the total ecoregion richness.

**Ecoregion name**	**Richness**	**Endemism**	**Urgent scenario**	**Moderate scenario**	**Most inclusive scenario**
Sierra Madre Occidental pine-oak forests	39	1 (2.6%)	0	0	5 (12.8%)
Sierra Madre Oriental pine-oak forests	39	3 (7.7%)	2 (5.1%)	7 (17.9%)	13 (33.3%)
Allegheny Highlands forests	27	0	0	0	0
Appalachian mixed mesophytic forests	58	1 (1.7%)	0	2 (3.4%)	3 (5.2%)
Appalachian-Blue Ridge forests	67	15 (22.4%)	0	1 (1.5%)	6 (9%)
Central U.S. hardwood forests	55	1 (1.8%)	0	0	1 (1.8%)
East Central Texas forests	29	0	0	1 (3.4%)	1 (3.4%)
Eastern forest-boreal transition	20	0	0	0	0
Eastern Great Lakes lowland forests	22	0	0	0	0
Gulf of St. Lawrence lowland forests	15	0	0	0	0
Mississippi lowland forests	35	0	0	0	0
New England-Acadian forests	20	0	0	0	0
Northeastern coastal forests	29	0	0	0	0
Ozark Mountain forests	39	3 (7.7%)	0	0	1 (2.6%)
Southeastern mixed forests	63	1 (1.6%)	0	2 (3.2%)	2 (3.2%)
Southern Great Lakes forests	33	0	0	0	0
Upper Midwest forest-savanna transition	22	0	0	0	0
Western Great Lakes forests	22	0	0	0	0
Willamette Valley forests	6	0	0	0	0
Alberta Mountain forests	4	0	0	0	0
Alberta-British Columbia foothills forests	5	0	0	0	0
Arizona Mountains forests	12	1 (8.3%)	0	0	1 (8.3%)
Atlantic coastal pine barrens	14	0	0	0	0
Blue Mountains forests	7	0	0	0	0
British Columbia mainland coastal forests	11	0	0	0	0
Cascade Mountains leeward forests	4	0	0	0	0
Central and Southern Cascades forests	20	3 (15%)	0	0	4 (20%)

Central British Columbia Mountain forests	4	0	0	0	0
Central Pacific coastal forests	19	0	0	0	2 (10.5%)
Colorado Rockies forests	7	0	0	0	0
Eastern Cascades forests	4	0	0	0	1 (25%)
Florida sand pine scrub	16	0	0	0	0
Fraser Plateau and Basin complex	5	0	0	0	0
Great Basin montane forests	4	0	0	0	0
Klamath-Siskiyou forests	15	0	0	0	0
Middle Atlantic coastal forests	49	2 (4.1%)	0	0	1 (2%)
North Central Rockies forests	9	2 (22.2%)	0	0	0
Northern California coastal forests	18	1 (5.6%)	0	0	1 (5.6%)
Northern Pacific coastal forests	4	0	0	0	0
Northern transitional alpine forests	5	0	0	0	1 (20%)
Okanagan dry forests	8	0	0	0	0
Piney Woods forests	35	0	0	0	0
Puget lowland forests	12	0	0	0	1 (8.3%)
Queen Charlotte Islands	1	0	0	0	0
Sierra Juarez and San Pedro Martir pine-oak forests	2	0	0	0	0
Sierra Nevada forests	13	3 (23.1%)	0	2 (15.4%)	4 (30.8%)
South Central Rockies forests	6	0	0	0	0
Southeastern conifer forests	53	4 (7.5%)	0	1 (1.9%)	4 (7.5%)
Wasatch and Uinta montane forests	7	0	0	0	0
Alaska Peninsula montane taiga	1	0	0	0	0
Central Canadian Shield forests	12	0	0	0	0
Cook Inlet taiga	1	0	0	0	0
Copper Plateau taiga	1	0	0	0	0
Eastern Canadian forests	12	0	0	0	0
Eastern Canadian Shield taiga	5	0	0	0	0
Interior Alaska-Yukon lowland taiga	1	0	0	0	0
Mid-Continental Canadian forests	6	0	0	0	1 (16.7%)
Midwestern Canadian Shield forests	7	0	0	0	0
Muskwa-Slave Lake forests	3	0	0	0	1 (33.3%)
Northern Canandian Shield taiga	3	0	0	0	0
Northern Cordillera forests	6	0	0	0	1 (16.7%)

Northwest Territories taiga	2	0	0	0	0
Southern Hudson Bay taiga	7	0	0	0	0
Yukon Interior dry forests	4	0	0	0	1 (25%)
Western Gulf coastal grasslands	32	0	0	2 (6.3%)	2 (6.3%)
California Central Valley grasslands	6	0	0	0	0
Canadian Aspen forests and parklands	9	0	0	0	0
Central and Southern mixed grasslands	19	0	0	0	0
Central forest-grasslands transition	44	0	0	0	0
Central tall grasslands	21	0	0	0	0
Edwards Plateau savanna	18	2 (11.1%)	0	0	2 (11.1%)
Flint Hills tall grasslands	15	0	0	0	0
Montana Valley and Foothill grasslands	8	0	0	0	0
Nebraska Sand Hills mixed grasslands	10	0	0	0	0
Northern mixed grasslands	15	0	0	0	0
Northern short grasslands	9	0	0	0	0
Northern tall grasslands	12	0	0	0	0
Palouse grasslands	8	0	0	0	0
Texas blackland prairies	33	3 (9.1%)	0	1 (3%)	3 (9.1%)
Western short grasslands	19	0	0	0	0
Alaska-St. Elias Range tundra	2	0	0	0	1 (50%)
Beringia lowland tundra	1	0	0	0	0
Beringia upland tundra	1	0	0	0	0
Interior Yukon-Alaska alpine tundra	1	0	0	0	0
Pacific Coastal Mountain icefields and tundra	5	0	0	0	1 (20%)
California coastal sage and chaparral	15	0	0	0	1 (6.7%)
California interior chaparral and woodlands	16	1 (6.3%)	0	0	2 (12.5%)
California montane chaparral and woodlands	14	1 (7.1%)	0	0	2 (14.3%)
Baja California desert	8	0	0	0	0
Central Mexican matorral	41	3 (7.3%)	5 (12.2%)	7 (17.1%)	13 (31.7%)
Chihuahuan desert	34	0	0	0	3 (8.8%)
Colorado Plateau shrublands	12	0	0	0	0
Great Basin shrub steppe	9	0	0	0	1 (11.1%)
Gulf of California xeric scrub	2	0	0	0	0
Meseta Central matorral	25	0	1 (4%)	4 (16%)	5 (20%)

Mojave desert	8	2 (25%)	0	2 (25%)	2 (25%)
Snake-Columbia shrub steppe	8	0	0	0	0
Sonoran desert	27	2 (7.4%)	0	0	0
Tamaulipan matorral	17	0	1 (5.9%)	2 (11.8%)	3 (17.6%)
Tamaulipan mezquital	29	0	0	1 (3.4%)	2 (6.9%)
Wyoming Basin shrub steppe	6	0	0	0	0
Araucaria moist forests	172	1 (0.6%)	3 (1.7%)	5 (2.9%)	6 (3.5%)
Atlantic Coast restingas	188	0	1 (0.5%)	2 (1.1%)	4 (2.1%)
Bahia coastal forests	95	0	0	0	3 (3.2%)
Bahia interior forests	145	1 (0.7%)	1 (0.7%)	2 (1.4%)	5 (3.4%)
Bolivian Yungas	53	24 (45.3%)	3 (5.7%)	7 (13.2%)	12 (22.6%)
Caatinga Enclaves moist forests	20	0	0	0	0
Caqueta moist forests	75	2 (2.7%)	1 (1.3%)	1 (1.3%)	1 (1.3%)
Catatumbo moist forests	3	0	0	0	0
Cauca Valley montane forests	58	20 (34.5%)	6 (10.3%)	14 (24.1%)	22 (37.9%)
Central American Atlantic moist forests	35	0	0	2 (5.7%)	3 (8.6%)
Central American montane forests	72	0	9 (12.5%)	28 (38.9%)	41 (56.9%)
Chiapas montane forests	46	1 (2.2%)	8 (17.4%)	12 (26.1%)	20 (43.5%)
Chimalapas montane forests	19	2 (10.5%)	3 (15.8%)	5 (26.3%)	7 (36.8%)
Chocó-Darién moist forests	137	24 (17.5%)	5 (3.6%)	9 (6.6%)	23 (16.8%)
Cordillera La Costa montane forests	40	26 (65%)	2 (5%)	8 (20%)	13 (32.5%)
Cordillera Oriental montane forests	82	25 (30.5%)	2 (2.4%)	11 (13.4%)	27 (32.9%)
Costa Rican seasonal moist forests	40	0	0	3 (7.5%)	4 (10%)
Cuban moist forests	37	2 (5.4%)	6 (16.2%)	21 (56.8%)	28 (75.7%)
Eastern Cordillera real montane forests	194	74 (38.1%)	14 (7.2%)	59 (30.4%)	84 (43.3%)
Eastern Panamanian montane forests	30	1 (3.3%)	0	0	2 (6.7%)
Guianan Highlands moist forests	98	17 (17.3%)	1 (1%)	1 (1%)	4 (4.1%)
Guianan moist forests	126	17 (13.5%)	0	0	3 (2.4%)
Gurupa varzea	31	0	0	0	1 (3.2%)
Hispaniolan moist forests	47	14 (29.8%)	23 (48.9%)	34 (72.3%)	40 (85.1%)
Iquitos varzea	127	1 (0.8%)	0	1 (0.8%)	2 (1.6%)
Isthmian-Atlantic moist forests	116	8 (6.9%)	4 (3.4%)	14 (12.1%)	26 (22.4%)
Isthmian-Pacific moist forests	97	4 (4.1%)	5 (5.2%)	9 (9.3%)	18 (18.6%)
Jamaican moist forests	19	7 (36.8%)	6 (31.6%)	14 (73.7%)	16 (84.2%)

Japurá-Solimoes-Negro moist forests	92	1 (1.1%)	0	0	1 (1.1%)
Juruá-Purus moist forests	63	0	0	0	0
Leeward Islands moist forests	11	0	1 (9.1%)	5 (45.5%)	5 (45.5%)
Madeira-Tapajós moist forests	50	0	0	0	0
Magdalena Valley montane forests	105	52 (49.5%)	12 (11.4%)	34 (32.4%)	57 (54.3%)
Magdalena-Urabá moist forests	46	3 (6.5%)	0	0	1 (2.2%)
Marajá varzea	53	0	0	0	1 (1.9%)
Maranhão Babaþu forests	26	0	0	0	0
Mato Grosso seasonal forests	49	0	0	0	0
Monte Alegre varzea	53	0	0	0	0
Napo moist forests	162	22 (13.6%)	0	3 (1.9%)	6 (3.7%)
Negro-Branco moist forests	67	3 (4.5%)	0	0	1 (1.5%)
Northeastern Brazil restingas	15	0	0	0	0
Northwestern Andean montane forests	213	111 (52.1%)	21 (9.9%)	59 (27.7%)	100 (46.9%)
Oaxacan montane forests	51	4 (7.8%)	8 (15.7%)	19 (37.3%)	24 (47.1%)
Orinoco Delta swamp forests	10	0	0	0	0
Pantanos de Centla	18	0	0	0	0
Guianan freshwater swamp forests	33	0	0	0	0
Alto Paranß Atlantic forests	285	0	6 (2.1%)	10 (3.5%)	12 (4.2%)
Pernambuco coastal forests	35	0	0	0	1 (2.9%)
Pernambuco interior forests	37	0	0	0	1 (2.7%)
Peruvian Yungas	116	64 (55.2%)	8 (6.9%)	24 (20.7%)	32 (27.6%)
Petén-Veracruz moist forests	101	9 (8.9%)	13 (12.9%)	31 (30.7%)	45 (44.6%)
Puerto Rican moist forests	17	11 (64.7%)	6 (35.3%)	11 (64.7%)	11 (64.7%)
Purus varzea	117	0	0	1 (0.9%)	2 (1.7%)
Purus-Madeira moist forests	64	0	0	0	0
Rio Negro campinarana	65	0	0	0	1 (1.5%)
Santa Marta montane forests	18	14 (77.8%)	5 (27.8%)	8 (44.4%)	9 (50%)
Serra do Mar coastal forests	233	4 (1.7%)	5 (2.1%)	6 (2.6%)	8 (3.4%)
Sierra de los Tuxtlas	40	5 (12.5%)	7 (17.5%)	9 (22.5%)	12 (30%)
Sierra Madre de Chiapas moist forests	44	2 (4.5%)	2 (4.5%)	9 (20.5%)	16 (36.4%)
Solimões-Japurá moist forests	131	0	0	1 (0.8%)	2 (1.5%)
South Florida rocklands	15	0	0	0	0
Southern Andean Yungas	49	4 (8.2%)	0	6 (12.2%)	11 (22.4%)

Southwest Amazon moist forests	168	14 (8.3%)	1 (0.6%)	2 (1.2%)	3 (1.8%)
Talamancan montane forests	122	37 (30.3%)	22 (18%)	40 (32.8%)	53 (43.4%)
Tapajós-Xingu moist forests	53	0	0	0	1 (1.9%)
Pantepui	52	28 (53.8%)	0	0	4 (7.7%)
Tocantins/Pindare moist forests	34	0	0	0	0
Trinidad and Tobago moist forests	2	0	0	1 (50%)	1 (50%)
Uatuma-Trombetas moist forests	89	0	0	0	1 (1.1%)
Ucayali moist forests	122	6 (4.9%)	0	1 (0.8%)	4 (3.3%)
Venezuelan Andes montane forests	55	35 (63.6%)	10 (18.2%)	26 (47.3%)	33 (60%)
Veracruz moist forests	58	4 (6.9%)	3 (5.2%)	13 (22.4%)	24 (41.4%)
Veracruz montane forests	27	2 (7.4%)	2 (7.4%)	6 (22.2%)	11 (40.7%)
Western Ecuador moist forests	85	10 (11.8%)	4 (4.7%)	11 (12.9%)	24 (28.2%)
Windward Islands moist forests	6	0	1 (16.7%)	2 (33.3%)	2 (33.3%)
Xingu-Tocantins-Araguaia moist forests	48	0	0	0	1 (2.1%)
Yucatán moist forests	35	0	0	1 (2.9%)	3 (8.6%)
Apure-Villavicencio dry forests	47	3 (6.4%)	1 (2.1%)	2 (4.3%)	2 (4.3%)
Atlantic dry forests	41	0	0	0	0
Bajío dry forests	33	0	0	3 (9.1%)	5 (15.2%)
Balsas dry forests	43	1 (2.3%)	2 (4.7%)	4 (9.3%)	12 (27.9%)
Bolivian montane dry forests	25	2 (8%)	0	1 (4%)	1 (4%)
Cauca Valley dry forests	6	0	0	0	0
Central American dry forests	36	0	2 (5.6%)	2 (5.6%)	3 (8.3%)
Dry Chaco	57	4 (7%)	1 (1.8%)	3 (5.3%)	3 (5.3%)
Chiapas Depression dry forests	33	0	2 (6.1%)	4 (12.1%)	8 (24.2%)
Chiquitano dry forests	54	0	0	0	0
Cuban dry forests	32	0	4 (12.5%)	16 (50%)	23 (71.9%)
Ecuadorian dry forests	13	0	0	0	1 (7.7%)
Hispaniolan dry forests	29	4 (13.8%)	13 (44.8%)	20 (69%)	24 (82.8%)
Jalisco dry forests	25	1 (4%)	1 (4%)	2 (8%)	3 (12%)
Jamaican dry forests	13	0	3 (23.1%)	9 (69.2%)	10 (76.9%)
Lara-Falcón dry forests	10	0	0	0	0
Lesser Antillean dry forests	6	0	1 (16.7%)	2 (33.3%)	2 (33.3%)
Magdalena Valley dry forests	19	0	0	0	0
Maracaibo dry forests	8	0	0	0	0

Marañón dry forests	4	0	1 (25%)	1 (25%)	1 (25%)
Panamanian dry forests	22	0	0	0	1 (4.5%)
Puerto Rican dry forests	6	0	1 (16.7%)	2 (33.3%)	2 (33.3%)
Sierra de la Laguna dry forests	2	0	0	0	0
Sinaloan dry forests	38	1 (2.6%)	1 (2.6%)	2 (5.3%)	7 (18.4%)
Sin· Valley dry forests	42	1 (2.4%)	1 (2.4%)	8 (19%)	12 (28.6%)
Southern Pacific dry forests	68	5 (7.4%)	9 (13.2%)	16 (23.5%)	26 (38.2%)
Tumbes-Piura dry forests	11	2 (18.2%)	0	1 (9.1%)	2 (18.2%)
Veracruz dry forests	26	0	0	2 (7.7%)	4 (15.4%)
Yucatán dry forests	19	1 (5.3%)	0	0	0
Bahamian pine mosaic	2	0	0	0	0
Belizian pine forests	14	0	0	0	0
Central American pine-oak forests	103	2 (1.9%)	15 (14.6%)	37 (35.9%)	52 (50.5%)
Cuban pine forests	23	0	1 (4.3%)	7 (30.4%)	14 (60.9%)
Hispaniolan pine forests	30	6 (20%)	11 (36.7%)	20 (66.7%)	24 (80%)
Sierra de la Laguna pine-oak forests	3	0	0	0	0
Sierra Madre del Sur pine-oak forests	13	0	0	0	0
Trans-Mexican Volcanic Belt pine-oak forests	78	8 (10.3%)	9 (11.5%)	25 (32.1%)	33 (42.3%)
Magellanic subpolar forests	12	2 (16.7%)	0	0	1 (8.3%)
Valdivian temperate forests	38	20 (52.6%)	6 (15.8%)	10 (26.3%)	17 (44.7%)
Beni savanna	29	0	0	0	0
Campos Rupestres montane savanna	113	0	2 (1.8%)	2 (1.8%)	3 (2.7%)
Cerrado	199	4 (2%)	4 (2%)	4 (2%)	5 (2.5%)
Guianan savanna	91	5 (5.5%)	0	0	2 (2.2%)
Humid Chaco	60	1 (1.7%)	1 (1.7%)	2 (3.3%)	2 (3.3%)
Llanos	39	1 (2.6%)	0	0	0
Uruguayan savanna	113	6 (5.3%)	1 (0.9%)	3 (2.7%)	6 (5.3%)
Espinal	29	1 (3.4%)	1 (3.4%)	1 (3.4%)	2 (6.9%)
Low Monte	10	0	1 (10%)	3 (30%)	4 (40%)
Humid Pampas	36	0	1 (2.8%)	2 (5.6%)	2 (5.6%)
Patagonian steppe	13	4 (30.8%)	0	4 (30.8%)	6 (46.2%)
Cuban wetlands	16	0	0	3 (18.8%)	8 (50%)
Enriquillo wetlands	9	0	0	2 (22.2%)	5 (55.6%)
Everglades	19	0	0	0	0

Guayaquil flooded grasslands	8	0	0	0	1 (12.5%)
Pantanal	54	0	0	0	0
Paraná flooded savanna	44	0	1 (2.3%)	2 (4.5%)	2 (4.5%)
Southern Cone Mesopotamian savanna	25	0	1 (4%)	1 (4%)	1 (4%)
Central Andean dry puna	14	5 (35.7%)	3 (21.4%)	5 (35.7%)	8 (57.1%)
Central Andean puna	24	4 (16.7%)	1 (4.2%)	4 (16.7%)	9 (37.5%)
Central Andean wet puna	21	11 (52.4%)	3 (14.3%)	7 (33.3%)	10 (47.6%)
Cordillera Central páramo	14	7 (50%)	3 (21.4%)	7 (50%)	7 (50%)
Cordillera de Merida páramo	3	1 (33.3%)	2 (66.7%)	2 (66.7%)	2 (66.7%)
Northern Andean páramo	89	37 (41.6%)	14 (15.7%)	40 (44.9%)	51 (57.3%)
Santa Marta páramo	2	0	2 (100%)	2 (100%)	2 (100%)
Southern Andean steppe	14	4 (28.6%)	2 (14.3%)	3 (21.4%)	4 (28.6%)
High Monte	11	1 (9.1%)	0	2 (18.2%)	4 (36.4%)
Chilean matorral	5	1 (20%)	0	0	0
Araya and Paria xeric scrub	1	0	0	0	0
Caatinga	49	0	0	0	1 (2%)
Caribbean shrublands	14	0	1 (7.1%)	4 (28.6%)	6 (42.9%)
Cuban cactus scrub	24	0	1 (4.2%)	12 (50%)	16 (66.7%)
Guajira-Barranquilla xeric scrub	8	1 (12.5%)	0	0	0
La Costa xeric shrublands	12	0	0	0	0
Motagua Valley thornscrub	23	0	0	0	0
Paraguana xeric scrub	2	0	0	0	0
San Lucan xeric scrub	2	0	0	0	0
Sechura desert	7	3 (42.9%)	0	0	2 (28.6%)
Tehuacán Valley matorral	29	3 (10.3%)	2 (6.9%)	7 (24.1%)	9 (31%)
Amazon-Orinoco-Southern Caribbean mangroves	14	0	0	1 (7.1%)	2 (14.3%)
Bahamian-Antillean mangroves	58	0	8 (13.8%)	25 (43.1%)	33 (56.9%)
Mesoamerican Gulf-Caribbean mangroves	52	0	0	0	5 (9.6%)
South American Pacific mangroves	34	0	0	0	2 (5.9%)
Southern Atlantic mangroves	59	0	1 (1.7%)	1 (1.7%)	1 (1.7%)
Southern Mesoamerican Pacific mangroves	54	0	0	0	3 (5.6%)
