# Supplementary material for: Land Use Explains the Distribution of Threatened New World Amphibians Better than Climate
Source: PLoS One. 2013 Apr 16;8(4):e60742. doi: 10.1371/journal.pone.0060742 (PMC3628793; doi:10.1371/journal.pone.0060742)
Supplement: Table S2 — Pearson correlation coefficient between the number of threatened amphibian species according to the three different scenarios (urgent, moderate and most inclusive), richness of amphibian species, endemism, two climatic axes, proportion of cover of villages and wildlands and the three axes of phylogenetic structure, all in residual form, which were used in the path analyses. (PDF) [file pone.0060742.s003.pdf]

Table S2. Pearson correlation coefficient between the number of threatened amphibian species according to the three different scenarios (urgent, moderate and most inclusive), richness of amphibian species, endemism, two climatic axes, proportion of cover of villages and wildlands and the three axes of phylogenetic structure, all in residual form, which were used in the path analyses.

	**Urgent**	**Moderate**	**Most Inclusive**
Richness	0.46	0.49	0.53
Endemism	0.63	0.73	0.78
Climate 1	-0.05	-0.05	-0.09
Climate 2	-0.50	-0.54	-0.58
Village	0.33	0.27	0.24
Wildlands	-	-0.14	-0.14
PCPS1	-0.14	-0.14	-0.15
PCPS2	0.20	0.21	0.21
PCPS3	-0.15	-0.19	-0.17
